# Uncovering the Fungal Community Composition of Alive and Dead *Posidonia oceanica* Matte

**DOI:** 10.1007/s00248-025-02492-6

**Published:** 2025-01-10

**Authors:** Sara Frasca, Annamaria Alabiso, Marco Maria D’Andrea, Luciana Migliore

**Affiliations:** 1 PhD Program in Evolutionary Biology and Ecology, Tor Vergata University of Rome, 00133 Rome, Italy; 2 Department of Biology, University of Rome Tor Vergata, 00133 Rome, Italy; 3 eCampus University, 22060 Novedrate (CO), Italy

**Keywords:** *Posidonia oceanica*, Alive matte, Dead matte, Fungal community, DNA metabarcoding

## Abstract

*Posidonia oceanica* retains a large amount of carbon within its belowground recalcitrant structure, the ‘matte,’ which is characterized by low oxygen availability and biodegradation. Fungi may play a pivotal role in carbon sequestration within the matte, even if little/no information is available. To fill this gap, we profiled fungal communities from the upper and lower layers of alive and dead matte, by using an ITS2-5.8S rDNA metabarcoding approach. The study was conducted in a shallow coastal stretch of the Aegean Sea (Crete). Then, 184 operational taxonomic units were identified, predominantly belonging to Ascomycota, in alive and dead matte. Nevertheless, their composition significantly differed: the host-specific *Posidoniomyces atricolor* was dominant in alive but not in dead matte, while fast-growing saprotrophs, potentially accelerating the decomposition rate, increased in dead matte. These findings lay the groundwork for future investigations on the possible increase of biodegradation under the changing environmental conditions.

**Supplementary Information:**

The online version contains supplementary material available at 10.1007/s00248-025-02492-6.

The Mediterranean seagrass *Posidonia oceanica* is regarded as an ecosystem engineer, able to stabilize sediments and sequester CO_2_ as *blue carbon* [1, 2]. It exhibits a remarkable carbon storage capacity through its dense leafy canopy and its extensive belowground structures known as ‘matte’ (Supplementary data, Fig. S1). Indeed, approximately 21% of the annual CO_2_ fixed by *P. oceanica* is sequestered within the matte [3], which can persist for thousands of years maintaining its integrity even after the meadows die [4, 5]. The long-term persistence is mainly due to the reduced biodegradation of the recalcitrant tissues and the anoxic conditions of the deep layers [6, 7], where the ability of microorganisms in decomposition processes is reduced, although their role is still understudied. Fungi are known to be able to decompose plant material via ligninolytic enzymes, breaking down recalcitrant compounds abundant in seagrass [8–10]; however, marine fungi are known to produce a wide range of hydrolytic and/or oxidative enzymes [11]. Moreover, recent studies revealed that fungal by-products—exudates and melanin—may reduce biodegradation and enhance soil carbon sequestration by stabilizing organic carbon [12–14]. However, environmental changes may modify fungal communities, reducing belowground carbon storage [15, 16].

We profiled the fungal community associated with *P. oceanica* matte in a living meadow (alive matte) and in nearby dead matte (dead matte), examining both the upper (at water–sediment interface) and lower layer (the deepest layer). Samples were collected in July 2021 at the Aegean Sea (35.335700 N, 25.281518 E) at 3–5 m depth, in three replicates (5-cm diameter cores, 8–13 cm in length, according to matte thicknesses), with the aim to determine fungal colonizer changes and their putative biodegradation activities. The matte samples were subjected to ITS2-5.8S rDNA metabarcoding by using an Illumina MiSeq platform. Sample preparation, extraction, sequencing, and bioinformatics analyses, according to Frasca et al. [17], are detailed in Supplementary data, Method section.

A total of 25,696 sequences (9583 in alive and 16,11 in dead matte), clustered in 184 OTUs with 97% sequence similarity (Supplementary data), were obtained after quality control/filtering steps. Nineteen OTUs had relative abundance > 3% and were differently distributed across matte samples (Fig. 1).

**Fig. 1:**
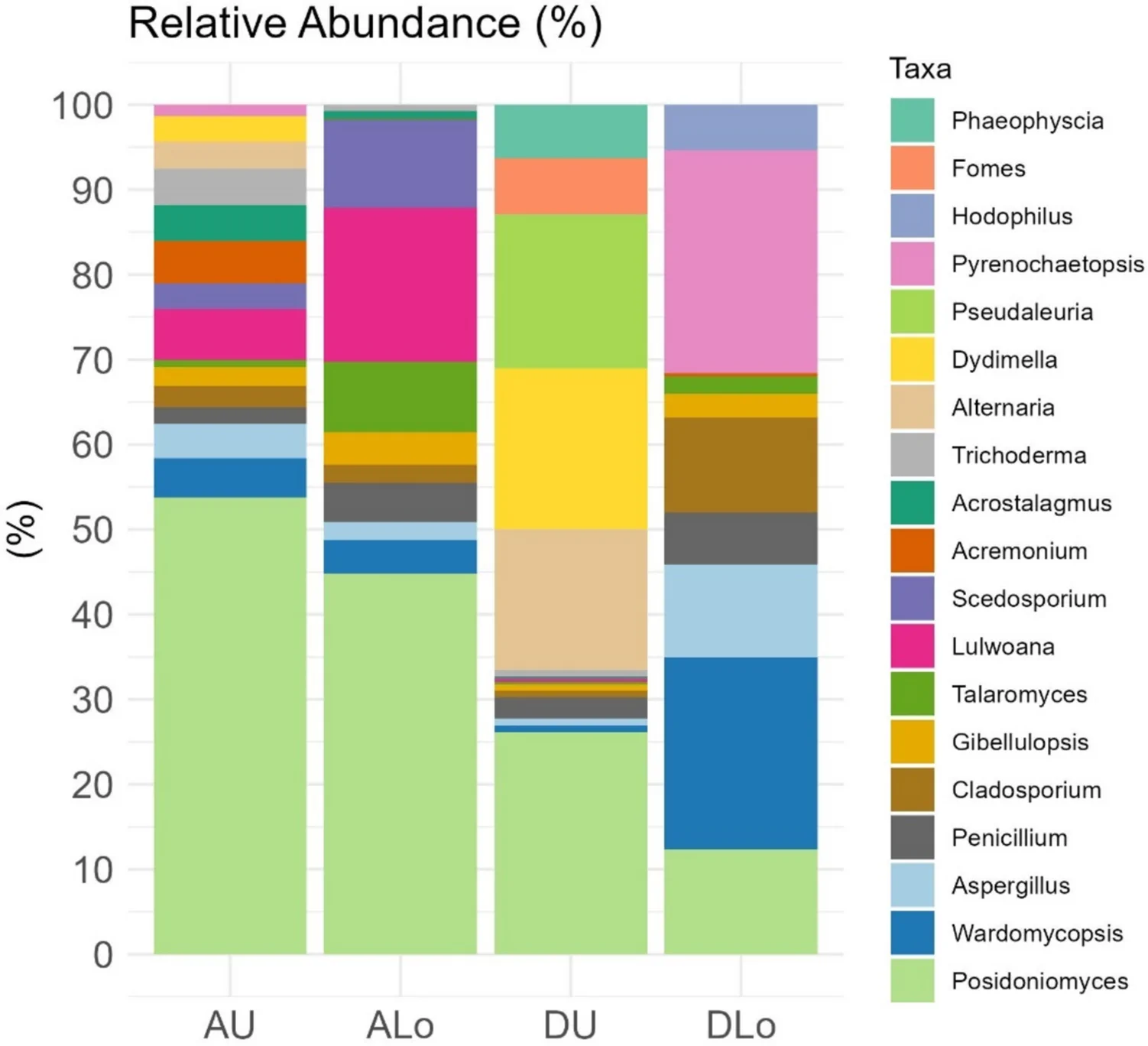
Dominant fungal genera in alive/dead *P. oceanica* matte upper/lower layers (A = alive; D = dead; U = upper; Lo = lower); the genera are stacked in the same order in the figure as in the key

Alpha-diversity resulted quite similar in all samples, with not significant differences between the upper and lower layers of the matte and between the alive and dead matte (Kruskal–Wallis test; *p* = ns; Table 1). The highest diversity was found in the upper layer of dead matte, which is directly exposed to oxygenated seawater, allowing the recruitment of a great deal of microorganisms from water column.

**Table 1:** Shannon index (*H*′) in alive/dead *P. oceanica* matte upper/lower layers

*P. oceanica ‘matte’*	*Layers*	*H′*
***Alive***	*Upper*	2.03 ± 0.64
	*Lower*	2.15 ± 0.76
***Dead***	*Upper*	2.41 ± 0.16
	*Lower*	2.06 ± 0.36

Conversely, β-diversity resulted significantly different between alive/dead matte fungal colonizers, but not between upper/lower layers (Table 2). This heterogeneity related also to functional groups identified by FUNGuild analysis, which was significantly different between alive/dead matte (two-way PERMANOVA test; *F* = 1.21; *p* = 0.03). As expected, saprotrophy was the dominant function (Fig. 2). The 131 OTUs classified based on their putative function through FUNGuild are reported in detail in Supplementary data, Table S2.

**Table 2:** Comparison of fungal colonizers in alive/dead *P. oceanica* matte upper/lower layers (PERMANOVA test, 999 permutations by using two distance metrics)

Comparison	Metrics	Test statistic	*p*-value
**Alive/dead**	Bray–Curtis	1.131	0.009
	UniFrac unweighted	1.470	0.027
**Alive upper/lower**	Bray–Curtis	1.016	ns
	UniFrac unweighted	0.842	ns
**Dead upper/lower**	Bray–Curtis	1.349	ns
	UniFrac unweighted	1.160	ns

**Fig. 2:**
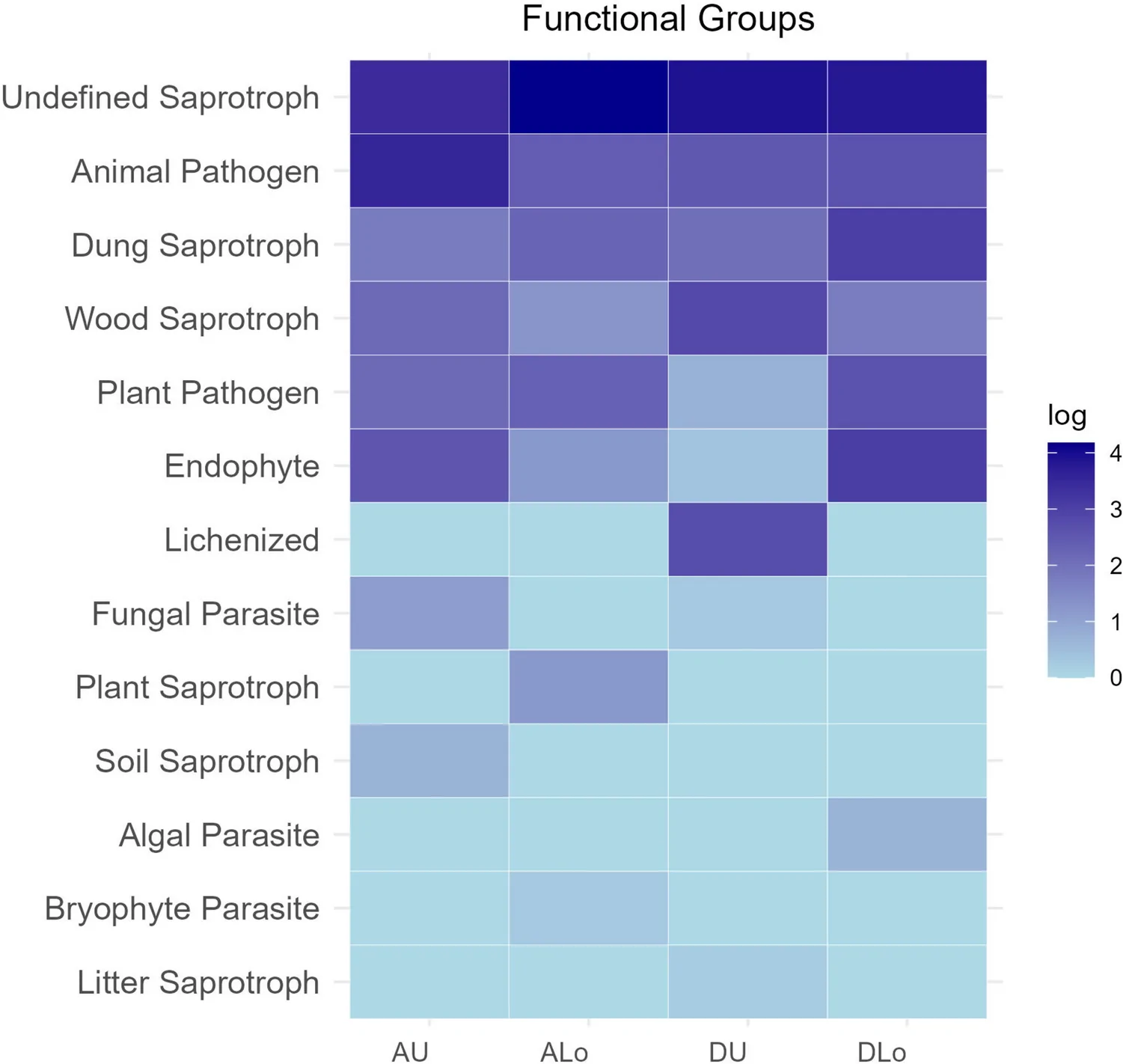
Functional groups of fungi in alive/dead and upper/lower layers of *P. oceanica* matte (A = alive; D = dead; U = upper; Lo = lower)

The taxonomic identification showed 97% of Ascomycota and only 3% of Basidiomycota. Ascomycota prefer generally aerobic environments, but their ability to utilize different compounds also under anoxic or sub-oxic conditions [18] allowed the colonization of both upper and lower layers, by displacing and/or increasing the mitochondrial number in oxygen deficiency conditions [19, 20]. This interpretation may explain the similarity of fungal community in the upper/lower samples, notwithstanding the possible oxygenation of the upper layer (due to living plants and hydrodynamics), absent in the lower layer. The Basidiomycota OTUs found in the matte were only 11 and exclusively present in dead matte (*Fomes inzengae* and *Hodophilus variabilipes* present > 2%, relative abundance); they were absent in alive matte. Some of them are efficient degraders of plant components and decaying material, putatively in line with the role of “late-stage” degraders in terrestrial systems [21]. Their absence or low relative abundance in matte may suggest a slow and weak decomposition rate.

The peculiar *P. oceanica* colonizer, *Posidoniomyces atricolor* [22, 23], was found in all samples: it was the most abundant taxa in alive upper layer (50%) but decreased in the alive lower (42%); the decrease was more pronounced in dead matte (from 25% in dead upper to 11% in dead lower layer). *P. atricolor* is a slow growing aerobic pleosporalean fungus, which seems to be specifically associated to *P. oceanica* roots, where it forms intracellular resting and storage structures, known as microsclerotia, supposed to be responsible for the putative dark septate endophyte colonization pattern [22–24]. *P. atricolor* hyphae and microsclerotia are melanized and, in soil, melanized structures protect against extreme temperatures, UV irradiation, and microbial degradation [13, 25, 26]. In a wider sense, this let us to hypothesize that root-symbiotic fungi may stabilize and sequester blue carbon in seagrass beds, as proposed by Vohník and Josefiová [24]. Furthermore, the highest relative abundance in both upper sections confirms that *P. atricolor* is more likely associated with *Posidonia* alive roots [27], the traces of its DNA may remain in dead roots and sediment, but progressively reducing after the plant death.

Beside *P. atricolor*, common colonizers of both alive and dead matte were the fast-growing saprotrophic *Aspergillus*, *Cladosporium*, *Penicillium*, and *Wardomycopsis*, showing the highest abundance in dead lower layer. There, they may contribute to matte degradation as, among these genera, there are degraders known to produce laccases, peroxidases, and tannases in the marine environment [9, 10]. *P. oceanica* has a high content of lignin which is refractory to bacterial digestion [8] and produces polyphenolic compounds, especially in stressful conditions [28, 29]. It is possible that part of the detected fast-growing saprobe DNA may belong to spores accumulated over time in the dead matte without significantly contributing to decomposition.

Some taxa were unique for specific matte layers: *Lulwoana* spp. were only found in alive matte and are supposed to be strictly associated with healthy *P. oceanica* roots [30], although their association and beneficial relationship with *P. oceanica* remains is still discussed [23]. Anyway, the order Lulworthiales, to which *Lulwoana* belongs, is known to include saprobic genera [31], as indicated by the FUNGuild analysis. Furthermore, a novel insight into the roles of fungi in marine environment comes from the occurrence of *Phaeophyscia orbicularis*, a putative lichenicolous fungus, only found in the upper layer of dead matte. Submarine lichens are very rare: among the few reports, the fungus *Chadefaudia corallinarum* was found associated with red algae on seagrass leaves in the Mediterranean Sea [32].

In conclusion, the taxonomic composition of fungal communities associated with alive/dead *P. oceanica* matte significantly differed. *P. atricolor* confirmed its strong association with the living meadows, while in dead matte an enrichment of fast-growing saprotrophic groups could contribute to its degradation. The slow matte breakdown rate grants the carbon burial over time, but environmental change could alter this equilibrium. By increasing oxygenation (due to high hydrodynamics) and consequent biodegradation rate, the *P. oceanica* matte carbon sink capacity may be hampered.

## Supplementary Information

Below is the link to the electronic supplementary material.

Supplementary file1 (PDF 898 KB)

## Data Availability

The fungal DNA sequences have been deposited in the Sequence Read Archive (SRA) database of NCBI under BioProject ID: PRJNA1186200.
