# Supplementary material for: Uncovering the Fungal Community Composition of Alive and Dead Posidonia oceanica Matte
Source: Microb Ecol. 2025 Jan 10;87(1):170. doi: 10.1007/s00248-025-02492-6 (PMC11717839; doi:10.1007/s00248-025-02492-6)
Supplement: Supplementary file 1 — Supplementary file1 (PDF 898 KB) [file 248_2025_2492_MOESM1_ESM.pdf]

Supplementary data

Uncovering the Fungal Community Composition of Alive and Dead *Posidonia oceanica* matte

Sara Frasca[1, 2], Annamaria Alabiso[2], Marco Maria D'Andrea[2], Luciana Migliore[2, 3]*

[1] PhD Program in Evolutionary Biology and Ecology, Tor Vergata University of Rome, 00133, Rome, Italy

[2] Department of Biology, University of Rome Tor Vergata, 00133, Rome, Italy

[3] eCampus University, 22060, Novedrate (CO), Italy

*Corresponding author: luciana.migliore@uniroma2.it; ORCID: 0000-0003-3554-3841

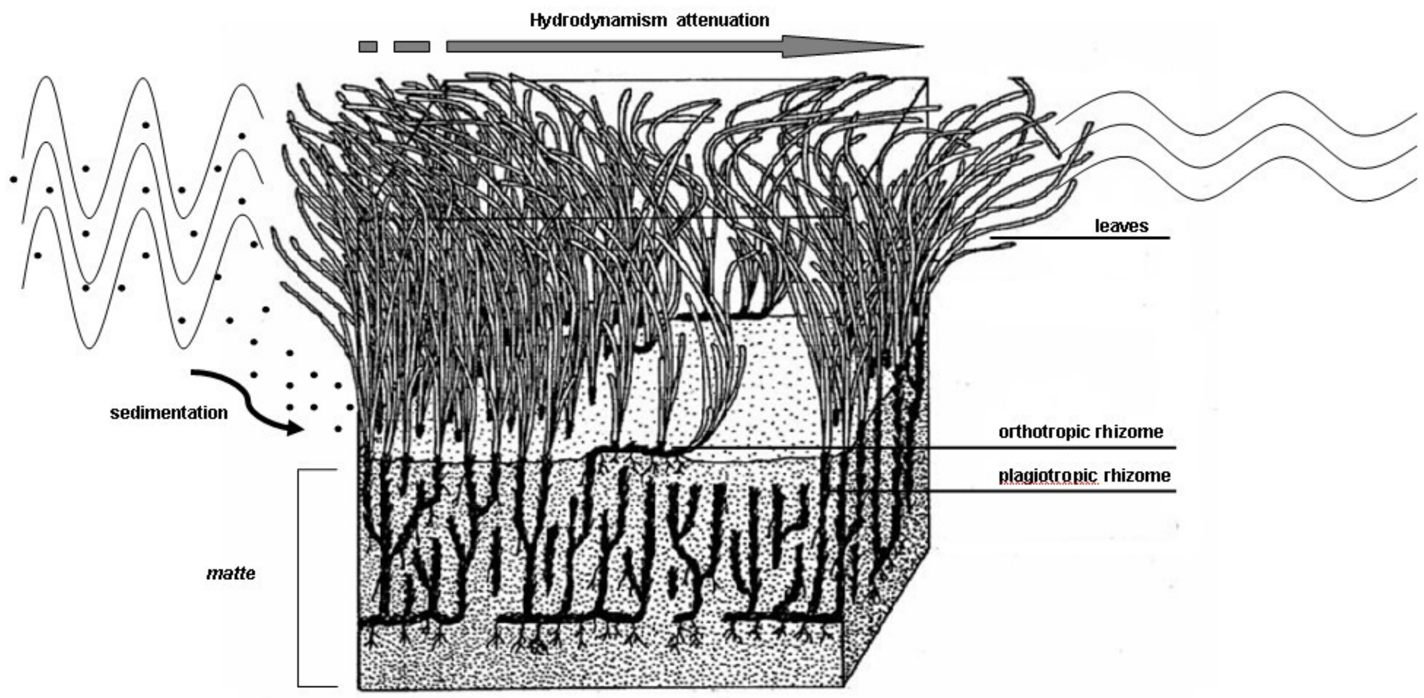

Fig. S1. Schematic representation of *Posidonia* meadows, showing the *matte*, rhizomes and leaves. (from Migliore et al., 2007; BMC Ecology, 7:6; https://doi.org/10.1186/1472-6785-7-6).

Methods section

To identify fungi with the potential to influence *Posidonia oceanica* matte degradation, we conducted ITS2-5.8S rDNA metabarcoding on three core replicates for each matte condition (live and dead) and each layer (upper and lower). This method was chosen for its ability to provide unprecedented taxonomic and functional information about fungi colonized *Posidonia* matte, as reported for soil fungi [1].

The study site was selected because it featured a *P. oceanica* meadow with thick matte, located 5 meters from a dead matte that lacked its vegetative cover.

Core samples, each with a 5 cm inner diameter, ranged from 8 to 13 cm in length, depending on the matte thickness. The entire matte thickness has been sampled, up to the underlying sand. Each core was sectioned to separate the two layers, and a 1 cm segment from each layer was collected for fungal analysis. In the upper layer, we observed root/shoot segments and sand, in the bottom layers sand were prevalent. Each segment was rinsed with 5 mL of washing solution (200 mM Tris-HCl pH 8, 10 mM EDTA, and 0.24% Triton X-100; [2]), vortexed in a 50 mL Falcon tube for 30 seconds. The solution was then centrifuged (20 min, 5000*g*) to obtain the microbial pellet, which was stored in 2 mL of transport solution (Tris 10 mM, EDTA 50 mM; [2]) until DNA extraction.

Fungal metagenomic DNA was extracted using the PowerSoil® DNA Isolation Kit (Mo Bio, Carlsbad, CA, USA) following the manufacturer's protocol. Extracted DNAs may belong to both spores and active mycelia, but spores may resist complete lysis, whereas mycelium typically lyses more easily. Hence, it is difficult to suppose that the DNA from the spores have been extracted, as our technique has not been dedicated to this (*i.e.*, it does not include the grounding in liquid nitrogen, using mortar with pestle and we did not subjected samples to spore germination and enrichment to make spore-DNA accessible during extraction. [3]). The ITS2-5.8S rDNA gene region of fungi was amplified by PCR using universal primers ITS3 (forward, 5′- GCATCGATGAAGAACGCAGC -3′) and ITS4 (reverse, 5′- TCCTCCGCTTATTGATATGC -3′; [4]). Including the 5.8S gene region improves fungal taxonomic identification, when databases are incomplete, as with marine fungi [5].

Each genomic DNA sample was sent for amplicon sequencing at BMR Genomics (Padova, Italy) using the Illumina MiSeq platform (Illumina, Inc., San Diego, CA, USA). Raw sequences have been deposited in the NCBI Sequence Read Archive under BioProject ID: PRJNA1186200.

Demultiplexed raw fungal sequences were processed using the Quantitative Insights into Microbial Ecology 2 (QIIME 2) platform (v2022.8). Primer sequences were removed using Cutadapt (v. 4.2; [6]). The raw sequences were then merged, quality-filtered, and denoised using the DADA2 plugin [7]. Taxonomic classification of the sequences was performed using Naïve Bayes classifiers trained on the UNITE database (v.8, 10.05.2021) by using a 99% threshold similarity levels [8]. The 99% similarity represents the highest correspondence level between the query and reference sequence, at the specific taxonomic level. We generated filtered tables and sequence files by excluding mitochondrial, chloroplast, and unclassified sequences. The ASVs (Amplicon Sequence Variants) were assigned to Operational Taxonomic Units (OTUs) at a 97% identity threshold, using the q2-vsearch plugin. Further manual processing was conducted to create the OTU table, resulting in the final taxonomic assignments reported in the Supplementary Data, Table S1. The taxonomic composition of the fungal communities (Fig. 1) included all OTUs with a relative abundance greater than 3%. The diversity indices (α- and β-diversity) were computed on fungal normalized dataset, obtained by the q2-srs plugin in QIIME 2. Graphs were generated in R Studio (v. 4.2.2). All OTUs taxonomically classified with QIIME 2 were submitted to FUNGuild v1.0 database for assigning the putative ecological functional annotations to each OTUs [9].

Methods References

1. Tedersoo L, Bahram M, Zinger L, Nilsson RH, Kennedy PG, Yang T, & Mikryukov V (2022) Best practices in metabarcoding of fungi: from experimental design to results. Mol Ecol 31:2769-2795.
2. Kadivar H & Stapleton AE (2003) Ultraviolet radiation alters maize phyllosphere bacterial diversity. Microb Ecol 45:353–361.
3. Corona Ramirez A, Bregnard D, Junier T, Cailleau G, Dorador C, Bindschedler S, Junier P (2023) Assessment of fungal spores and spore-like diversity in environmental samples by targeted lysis. BMC microbiol 23:68.

4. White TJ, Bruns T, Lee SJWT, & Taylor J. (1990) Amplification and direct sequencing of fungal ribosomal RNA genes for phylogenetics. PCR Protocols. A Guide to Methods and Applications 18:315-322.
5. Heeger F, Wurzbacher C, Bourne EC, Mazzoni CJ, & Monaghan MT (2019) Combining the 5.8 S and ITS2 to improve classification of fungi. Methods Ecol Evol 10:1702-1711.
6. Martin M (2011) Cutadapt removes adapter sequences from high-throughput sequencing reads. EMBnet j 17:10-12.
7. Callahan BJ, McMurdie PJ, Rosen MJ, Han AW, Johnson AJA, & Holmes SP (2016) DADA2: High-resolution sample inference from Illumina amplicon data. Nat Methods 13:581-583.
8. Kõljalg U, Nilsson RH, Abarenkov K, Tedersoo L, Taylor AF, Bahram M, & Larsson KH (2013) Towards a unified paradigm for sequence-based identification of fungi.
9. Nguyen NH, Song Z, Bates ST, Branco S, Tedersoo L, Menke J, & Kennedy PG (2016) FUNGuild: an open annotation tool for parsing fungal community datasets by ecological guild. Fungal Ecol 20:241-248.

Table S1. Fungal 97% OTUs identification in both alive and dead *P. oceanica* matte detected by using the Naïve Bayes classifiers trained on the UNITE database (v.8, 10.05.2021) by using a 99% threshold similarity levels.

* Conf.= confidence. The confidence value refers to the last classified taxonomic level. It ranges from 0 to 1; a value close to 1 suggests the best assignment.

**OTU**	**Kingdom**	**Phylum**	**Class**	**Order**	**Family**	**Genus**	**Species**	**Conf. ***
**OTU001**	Fungi	Ascomycota	Sordariomycetes	Hypocreales	Bionectriaceae	*Clonostachys*	*Clonostachys_rosea*	0,99
**OTU002**	Fungi	Ascomycota	Eurotiomycetes	Eurotiales	Aspergillaceae	*Aspergillus*	NA	1,00
**OTU003**	Fungi	Ascomycota	Eurotiomycetes	Eurotiales	Aspergillaceae	*Aspergillus*	*Aspergillus penicillioides*	0,99
**OTU004**	Fungi	Ascomycota	Eurotiomycetes	Eurotiales	Aspergillaceae	*Aspergillus*	NA	1,00
**OTU005**	Fungi	Ascomycota	Eurotiomycetes	Eurotiales	Aspergillaceae	*Aspergillus*	NA	1,00
**OTU006**	Fungi	Ascomycota	Eurotiomycetes	Eurotiales	Aspergillaceae	*Penicillium*	NA	0,88
**OTU007**	Fungi	Ascomycota	Eurotiomycetes	Eurotiales	Aspergillaceae	*Penicillium*	*Penicillium georgiense*	1,00
**OTU008**	Fungi	Ascomycota	Eurotiomycetes	Eurotiales	Aspergillaceae	*Penicillium*	*Penicillium adametzioides*	1,00
**OTU009**	Fungi	Ascomycota	Eurotiomycetes	Eurotiales	Aspergillaceae	*Aspergillus*	NA	1,00
**OTU010**	Fungi	Ascomycota	Eurotiomycetes	Onygenales	Onygenaceae	*Auxarthron*	*Auxarthron chlamydosporum*	1,00
**OTU011**	Fungi	Ascomycota	Dothideomycetes	Pleosporales	Sporormiaceae	*Sparticola*	NA	1,00
**OTU012**	Fungi	Ascomycota	Saccharomycetes	Saccharomycetales	Saccharomycetales_fam_Incertae_sedis	*Candida*	*Candida norvegica*	1,00
**OTU013**	Fungi	Ascomycota	Dothideomycetes	Pleosporales	Thyridariaceae	*Roussoella*	NA	1,00
**OTU014**	Fungi	Ascomycota	Eurotiomycetes	Onygenales	Onygenaceae	*Myriodontium*	*Myriodontium keratinophilum*	0,91
**OTU015**	Fungi	Ascomycota	Dothideomycetes	Pleosporales	Sporormiaceae	*Preussia*	NA	0,96
**OTU016**	Fungi	Ascomycota	Dothideomycetes	Pleosporales	Phaeosphaeriaceae	NA	NA	1,00
**OTU017**	Fungi	Ascomycota	Dothideomycetes	Capnodiales	Cladosporiaceae	*Cladosporium*	NA	0,99
**OTU018**	Fungi	Ascomycota	Sordariomycetes	Glomerellales	Plectosphaerellaceae	*Gibellulopsis*	NA	1,00
**OTU019**	Fungi	Ascomycota	Dothideomycetes	Pleosporales	Pyrenochaetopsidaceae	*Pyrenochaetopsis*	NA	1,00
**OTU020**	Fungi	Ascomycota	Dothideomycetes	Pleosporales	Dictyosporiaceae	*Pseudoconiothyrium*	*Pseudoconiothyrium broussonetiae*	0,76
**OTU021**	Fungi	Ascomycota	Saccharomycetes	Saccharomycetales	Saccharomycopsidaceae	*Saccharomycopsis*	*Saccharomycopsis amapae*	0,85
**OTU022**	Fungi	Basidiomycota	Agaricomycetes	Agaricales	Psathyrellaceae	*Coprinellus*	*Coprinellus verrucispermus*	0,96
**OTU023**	Fungi	Mucoromycota	Mucoromycetes	Mucorales	Rhizopodaceae	*Rhizopus*	*Rhizopus arrhizus*	1,00

OTU	Kingdom	Phylum	Class	Order	Family	Genus	Species	Conf. *
**OTU024**	Fungi	Ascomycota	Sordariomycetes	Microascales	Microascaceae	*Microascus*	NA	0,71
**OTU025**	Fungi	Ascomycota	Dothideomycetes	Pleosporales	Sporormiaceae	*Westerdykella*	*Westerdykella reniformis*	1,00
**OTU026**	Fungi	Ascomycota	Sordariomycetes	Microascales	Graphiaceae	*Graphium*	NA	0,92
**OTU027**	Fungi	Ascomycota	Sordariomycetes	Hypocreales	Hypocreales_fam_Incertae_sedis	*Acremonium*	*Acremonium spinosum*	0,99
**OTU028**	Fungi	Ascomycota	Eurotiomycetes	Eurotiales	Trichocomaceae	*Talaromyces*	*Talaromyces albobiverticillius*	0,99
**OTU029**	Fungi	Ascomycota	Eurotiomycetes	Eurotiales	Trichocomaceae	*Talaromyces*	*Talaromyces ucrainicus*	0,95
**OTU030**	Fungi	Ascomycota	Eurotiomycetes	Eurotiales	Trichocomaceae	*Talaromyces*	*Talaromyces clemensii*	0,80
**OTU031**	Fungi	Ascomycota	Eurotiomycetes	Eurotiales	Trichocomaceae	*Talaromyces*	*Talaromyces rubicundus*	0,93
**OTU032**	Fungi	Ascomycota	Eurotiomycetes	Eurotiales	Trichocomaceae	*Talaromyces*	*Talaromyces rubicundus*	0,83
**OTU033**	Fungi	Ascomycota	Saccharomycetes	Saccharomycetales	Debaryomycetaceae	*Schwanniomyces*	*Schwanniomyces pseudopolymorphus*	1,00
**OTU034**	Fungi	Ascomycota	Sordariomycetes	Microascales	Microascaceae	*Kernia*	*Kernia columnaris*	1,00
**OTU035**	Fungi	Ascomycota	Leotiomycetes	Helotiales	Vibrisseaceae	*Phialocephala*	*Phialocephala humicola*	1,00
**OTU036**	Fungi	Ascomycota	Sordariomycetes	Glomerellales	Plectosphaerellaceae	*Acrostalagmus*	*Acrostalagmus luteoalbus*	1,00
**OTU037**	Fungi	Ascomycota	Sordariomycetes	Hypocreales	Bionectriaceae	*Clonostachys*	NA	1,00
**OTU038**	Fungi	Ascomycota	Sordariomycetes	Hypocreales	Cordycipitaceae	*Beauveria*	NA	1,00
**OTU039**	Fungi	Basidiomycota	Agaricomycetes	Phallales	Phallaceae	*Phallus*	*Phallus impudicus*	1,00
**OTU040**	Fungi	Ascomycota	Dothideomycetes	Pleosporales	Cucurbitariaceae	*Pyrenochaetopsis*	*Pyrenochaetopsis leptospora*	1,00
**OTU041**	Fungi	Ascomycota	Dothideomycetes	Pleosporales	Didymosphaeriaceae	*Paraconiothyrium*	NA	0,91
**OTU042**	Fungi	Ascomycota	Dothideomycetes	Pleosporales	Didymosphaeriaceae	*Paraconiothyrium*	*Paraconiothyrium variabile*	1,00
**OTU043**	Fungi	Ascomycota	Dothideomycetes	Pleosporales	Pleosporaceae	*Paradendryphiella*	*Paradendryphiella arenariae*	0,77
**OTU044**	Fungi	Ascomycota	Dothideomycetes	Pleosporales	Pleosporaceae	*Alternaria*	NA	1,00
**OTU045**	Fungi	Basidiomycota	Agaricomycetes	Boletales	Coniophoraceae	*Coniophora*	NA	1,00
**OTU046**	Fungi	Ascomycota	Sordariomycetes	Microascales	Halosphaeriaceae	*Corollospora*	NA	0,72
**OTU047**	Fungi	Basidiomycota	Tremellomycetes	Tremellales	Bulleribasidiaceae	*Vishniacozyma*	*Vishniacozyma heimaeyensis*	1,00
**OTU048**	Fungi	Ascomycota	Eurotiomycetes	Eurotiales	Aspergillaceae	*Penicillium*	*Penicillium nalgiovense*	0,81

OTU	Kingdom	Phylum	Class	Order	Family	Genus	Species	Conf. *
**OTU049**	Fungi	Ascomycota	Eurotiomycetes	Eurotiales	Aspergillaceae	*Aspergillus*	*Aspergillus fumisynnematus*	0,92
**OTU050**	Fungi	Ascomycota	Eurotiomycetes	Eurotiales	Aspergillaceae	NA	NA	0,96
**OTU051**	Fungi	Ascomycota	Eurotiomycetes	Eurotiales	Aspergillaceae	*Penicillium*	*Penicillium brevicompactum*	0,96
**OTU052**	Fungi	Ascomycota	Eurotiomycetes	Eurotiales	Aspergillaceae	*Penicillium*	*Penicillium pimiteouiense*	0,85
**OTU053**	Fungi	Ascomycota	Eurotiomycetes	Eurotiales	Aspergillaceae	*Aspergillus*	*Aspergillus versicolor*	0,94
**OTU054**	Fungi	Ascomycota	NA	NA	NA	NA	NA	1,00
**OTU055**	Fungi	Ascomycota	Eurotiomycetes	Eurotiales	Aspergillaceae	*Penicillium*	NA	0,99
**OTU056**	Fungi	Ascomycota	Eurotiomycetes	Eurotiales	Aspergillaceae	*Aspergillus*	*Aspergillus spelaeus*	0,99
**OTU057**	Fungi	Ascomycota	Eurotiomycetes	Eurotiales	Aspergillaceae	*Penicillium*	*Penicillium menonorum*	0,96
**OTU058**	Fungi	Ascomycota	Eurotiomycetes	Eurotiales	Aspergillaceae	*Penicillium*	*Penicillium ubiquetum*	0,89
**OTU059**	Fungi	Ascomycota	Eurotiomycetes	Eurotiales	Aspergillaceae	*Penicillium*	NA	0,76
**OTU060**	Fungi	Ascomycota	Eurotiomycetes	Eurotiales	Aspergillaceae	*Penicillium*	NA	1,00
**OTU061**	Fungi	Ascomycota	Eurotiomycetes	Eurotiales	Aspergillaceae	*Aspergillus*	NA	1,00
**OTU062**	Fungi	Ascomycota	Eurotiomycetes	Onygenales	Gymnoascaceae	*Gymnoascus*	*Gymnoascus udagawae*	0,78
**OTU063**	Fungi	Ascomycota	Sordariomycetes	Xylariales	Apiosporaceae	*Arthrinium*	NA	1,00
**OTU064**	Fungi	Ascomycota	Sordariomycetes	Xylariales	Apiosporaceae	*Arthrinium*	*Arthrinium rasikravindrae*	1,00
**OTU065**	Fungi	Ascomycota	Dothideomycetes	Pleosporales	Sporormiaceae	*Sporormiella*	*Sporormiella minimoides*	1,00
**OTU066**	Fungi	Ascomycota	Eurotiomycetes	Onygenales	Onygenaceae	*Auxarthron*	*Auxarthron ostraviense*	0,97
**OTU067**	Fungi	Ascomycota	Sordariomycetes	Sordariales	Cephalothecaceae	*Phialemonium*	*Phialemonium inflatum*	0,93
**OTU068**	Fungi	Ascomycota	Sordariomycetes	Xylariales	Xylariaceae	*Ascotricha*	*Ascotricha erinacea*	1,00
**OTU069**	Fungi	Ascomycota	Sordariomycetes	Hypocreales	Nectriaceae	*Paracremonium*	NA	0,93
**OTU070**	Fungi	Ascomycota	Sordariomycetes	Hypocreales	Nectriaceae	*Fusarium*	*Fusarium nematophilum*	1,00
**OTU071**	Fungi	Ascomycota	Sordariomycetes	Hypocreales	Nectriaceae	*Rectifusarium*	*Rectifusarium ventricosum*	1,00
**OTU072**	Fungi	Ascomycota	Dothideomycetes	Pleosporales	Didymellaceae	*Didymella*	NA	0,74
**OTU073**	Fungi	Ascomycota	Dothideomycetes	Pleosporales	Didymellaceae	NA	NA	0,83
**OTU074**	Fungi	Ascomycota	Dothideomycetes	Capnodiales	Capnodiaceae	*Antennariella*	*Antennariella placitae*	1,00
**OTU075**	Fungi	Ascomycota	Eurotiomycetes	Onygenales	Gymnoascaceae	NA	NA	0,91

**OTU**	**Kingdom**	**Phylum**	**Class**	**Order**	**Family**	**Genus**	**Species**	**Conf. ***
**OTU076**	Fungi	Ascomycota	Eurotiomycetes	Eurotiales	Aspergillaceae	*Aspergillus*	*Aspergillus puniceus*	0,82
**OTU077**	Fungi	Ascomycota	Eurotiomycetes	Eurotiales	Aspergillaceae	*Aspergillus*	*Aspergillus versicolor*	0,99
**OTU078**	Fungi	Ascomycota	Dothideomycetes	Pleosporales	Occultibambusaceae	*Brunneofusispora*	*Brunneofusispora sinensis*	0,85
**OTU079**	Fungi	Ascomycota	Dothideomycetes	Pleosporales	NA	*NA*	*NA*	0,96
**OTU080**	Fungi	Ascomycota	Dothideomycetes	Pleosporales	Occultibambusaceae	*Brunneofusispora*	*Brunneofusispora sinensis*	0,99
**OTU081**	Fungi	Ascomycota	Saccharomycetes	Saccharomycetales	Lipomycetaceae	*Lipomyces*	*Lipomyces tetrasporus*	1,00
**OTU082**	Fungi	Ascomycota	Saccharomycetes	Saccharomycetales	Lipomycetaceae	*Lipomyces*	*Lipomyces tetrasporus*	1,00
**OTU083**	Fungi	Ascomycota	Sordariomycetes	Hypocreales	Hypocreaceae	*Trichoderma*	NA	1,00
**OTU084**	Fungi	Ascomycota	Eurotiomycetes	Onygenales	Onygenales_fam_Incertae_sedis	*Chrysosporium*	*Chrysosporium lobatum*	1,00
**OTU085**	Fungi	Ascomycota	Eurotiomycetes	Onygenales	Onygenales_fam_Incertae_sedis	*Chrysosporium*	*Chrysosporium lobatum*	1,00
**OTU086**	Fungi	Ascomycota	Sordariomycetes	Hypocreomycetidae	Plectosphaerellaceae	*Plectosphaerellaceae*	NA	1,00
**OTU087**	Fungi	Ascomycota	Sordariomycetes	Xylariales	Hyponectriaceae	*Monographella*	*Monographella nivalis*	0,84
**OTU088**	Fungi	Basidiomycota	Agaricomycetes	Agaricales	Clavariaceae	*Hodophilus*	*Hodophilus variabilipes*	0,76
**OTU089**	Fungi	Ascomycota	Saccharomycetes	Saccharomycetales	Saccharomycetales_fam_Incertae_sedis	*Candida*	*Candida norvegica*	1,00
**OTU090**	Fungi	Basidiomycota	Agaricomycetes	Agaricales	Lycoperdaceae	*Bovista*	NA	1,00
**OTU091**	Fungi	Basidiomycota	Agaricomycetes	Agaricales	Lycoperdaceae	*Bovista*	*Bovista aestivalis*	1,00
**OTU092**	Fungi	Ascomycota	Eurotiomycetes	Onygenales	Onygenales_fam_Incertae_sedis	*Chrysosporium*	*Chrysosporium pseudomerdarium*	0,88
**OTU093**	Fungi	Ascomycota	Leotiomycetes	Thelebolales	Pseudeurotiaceae	*Geomyces*	NA	0,96
**OTU094**	Fungi	Ascomycota	Sordariomycetes	Xylariales	Diatrypaceae	*Eutypa*	*Eutypa tetragona*	0,98
**OTU095**	Fungi	Ascomycota	Sordariomycetes	Hypocreales	Stachybotryaceae	*Stachybotrys*	*Stachybotrys chartarum*	1,00
**OTU096**	Fungi	Ascomycota	Sordariomycetes	Hypocreales	Hypocreaceae	*Hypomyces*	NA	0,93
**OTU097**	Fungi	Ascomycota	Dothideomycetes	Pleosporales	Lophiostomataceae	*Lophiostoma*	NA	0,80
**OTU098**	Fungi	Ascomycota	Sordariomycetes	Hypocreales	Stachybotryaceae	*Myrothecium*	*Myrothecium roridum*	0,82
**OTU099**	Fungi	Ascomycota	Sordariomycetes	Hypocreales	Stachybotryaceae	*Striaticonidium*	*Striaticonidium cinctum*	0,98
**OTU100**	Fungi	Ascomycota	Dothideomycetes	Pleosporales	Thyridariaceae	*Roussoella*	NA	0,88
**OTU101**	Fungi	Ascomycota	Dothideomycetes	Pleosporales	Thyridariaceae	*Roussoella*	*Roussoella neopustulans*	1,00
**OTU102**	Fungi	Ascomycota	Dothideomycetes	Pleosporales	Thyridariaceae	*Parathyridaria*	NA	1,00
**OTU103**	Fungi	Ascomycota	Dothideomycetes	Pleosporales	Thyridariaceae	*Parathyridaria*	NA	1,00
**OTU104**	Fungi	Ascomycota	Eurotiomycetes	Eurotiales	Aspergillaceae	*Penicillium*	NA	1,00

OTU	Kingdom	Phylum	Class	Order	Family	Genus	Species	Conf. *
**OTU105**	Fungi	Ascomycota	Eurotiomycetes	Eurotiales	Aspergillaceae	*Penicillium*	NA	1,00
**OTU106**	Fungi	Ascomycota	Sordariomycetes	Hypocreales	Nectriaceae	*Volutella*	NA	0,83
**OTU107**	Fungi	Ascomycota	Sordariomycetes	Lulworthiales	Lulworthiaceae	*Lulwoana*	NA	1,00
**OTU108**	Fungi	Ascomycota	Sordariomycetes	Lulworthiales	Lulworthiaceae	*Lulwoana*	NA	1,00
**OTU109**	Fungi	Ascomycota	Leotiomycetes	Helotiales	Helotiales_fam_Incertae_sedis	*Xylogone*	NA	1,00
**OTU110**	Fungi	Ascomycota	Leotiomycetes	Helotiales	Helotiaceae	*Scytalidium*	NA	1,00
**OTU111**	Fungi	Ascomycota	Lecanoromycetes	Caliciales	Physciaceae	*Phaeophyscia*	*Phaeophyscia orbicularis*	1,00
**OTU112**	Fungi	Basidiomycota	Agaricomycetes	Polyporales	Polyporaceae	*Fomes*	*Fomes inzengae*	1,00
**OTU113**	Fungi	Ascomycota	Sordariomycetes	Hypocreales	Stachybotryaceae	*Albifimbria*	NA	1,00
**OTU114**	Fungi	Ascomycota	Sordariomycetes	Hypocreales	Hypocreaceae	*Trichoderma*	NA	1,00
**OTU115**	Fungi	Ascomycota	Sordariomycetes	Hypocreales	Hypocreaceae	*Trichoderma*	*Trichoderma koningii*	0,85
**OTU116**	Fungi	Ascomycota	Sordariomycetes	Hypocreales	Hypocreaceae	*Trichoderma*	*Trichoderma albolutescens*	0,95
**OTU117**	Fungi	Ascomycota	Sordariomycetes	Hypocreales	Hypocreaceae	*Trichoderma*	NA	1,00
**OTU118**	Fungi	Ascomycota	Sordariomycetes	Hypocreales	Hypocreaceae	*Trichoderma*	*Trichoderma koningii*	0,75
**OTU119**	Fungi	Ascomycota	Dothideomycetes	Pleosporales	Biatriosporaceae	*Biatriospora*	*Biatriospora antibiotica*	1,00
**OTU120**	Fungi	Ascomycota	Eurotiomycetes	Onygenales	Onygenaceae	*Aphanoascus*	*Aphanoascus keratinophilus*	1,00
**OTU121**	Fungi	Ascomycota	Eurotiomycetes	Onygenales	Onygenaceae	*Aphanoascus*	*Aphanoascus keratinophilus*	0,98
**OTU122**	Fungi	Ascomycota	Sordariomycetes	Hypocreales	NA	NA	NA	0,75
**OTU123**	Fungi	Ascomycota	Sordariomycetes	Hypocreales	Hypocreales_fam_Incertae_sedis	*Acremonium*	*Acremonium hennebertii*	0,94
**OTU124**	Fungi	Ascomycota	Pezizomycetes	Pezizales	Pyronemataceae	*Pseudaleuria*	NA	1,00
**OTU125**	Fungi	Ascomycota	Sordariomycetes	Xylariales	Diatrypaceae	*Eutypa*	*Eutypa flavovirens*	1,00
**OTU126**	Fungi	Ascomycota	Sordariomycetes	Hypocreales	NA	NA	NA	0,86
**OTU127**	Fungi	Ascomycota	Dothideomycetes	Capnodiales	Cladosporiaceae	*Cladosporium*	NA	0,91
**OTU128**	Fungi	Ascomycota	Dothideomycetes	Capnodiales	Cladosporiaceae	*Cladosporium*	NA	0,91
**OTU129**	Fungi	Ascomycota	Eurotiomycetes	Eurotiales	Aspergillaceae	*Aspergillus*	NA	1,00
**OTU130**	Fungi	Ascomycota	Sordariomycetes	Sordariales	Chaetomiaceae	*Humicola*	*Humicola parvispora*	1,00
**OTU131**	Fungi	Ascomycota	Dothideomycetes	Pleosporales	Periconiaceae	*Periconia*	NA	0,98
**OTU132**	Fungi	Ascomycota	Pezizomycetes	Pezizales	Pyronemataceae	*Heydenia*	*Heydenia alpina*	0,81

OTU	Kingdom	Phylum	Class	Order	Family	Genus	Species	Conf. *
**OTU133**	Fungi	Ascomycota	Sordariomycetes	Microascales	Microascaceae	*Cephalotrichum*	*Cephalotrichum asperulum*	0,98
**OTU134**	Fungi	Ascomycota	Sordariomycetes	Microascales	Microascaceae	*Gamsia*	*Gamsia simplex*	0,99
**OTU135**	Fungi	Ascomycota	Sordariomycetes	Hypocreales	Clavicipitaceae	*Metarhizium*	*Metarhizium robertsii*	0,90
**OTU136**	Fungi	Ascomycota	Sordariomycetes	Hypocreales	Hypocreales_fam_Incertae_sedis	*Acremonium*	*Acremonium dichromosporum*	0,85
**OTU137**	Fungi	Basidiomycota	Wallemiomycetes	Wallemiales	Wallemiaceae	*Wallemia*	*Wallemia sebi*	0,93
**OTU138**	Fungi	Basidiomycota	Wallemiomycetes	Wallemiales	Wallemiaceae	*Wallemia*	*NA*	1,00
**OTU139**	Fungi	Ascomycota	Sordariomycetes	Hypocreales	Hypocreales_fam_Incertae_sedis	*Acremonium*	*Acremonium cavaraeanum*	1,00
**OTU140**	Fungi	Ascomycota	Dothideomycetes	Pleosporales	NA	NA	NA	0,99
**OTU141**	Fungi	Ascomycota	Dothideomycetes	Pleosporales	Phaeosphaeriaceae	*Setophaeosphaeria*	*Setophaeosphaeria badalingensis*	0,99
**OTU142**	Fungi	Ascomycota	Dothideomycetes	Pleosporales	Cucurbitariaceae	*Pyrenochaeta*	NA	0,81
**OTU143**	Fungi	Ascomycota	Sordariomycetes	Sordariales	Chaetomiaceae	*Botryotrichum*	*Botryotrichum spirotrichum*	0,80
**OTU144**	Fungi	Ascomycota	Sordariomycetes	Sordariales	Chaetomiaceae	*Humicola*	*Humicola grisea*	0,95
**OTU145**	Fungi	Ascomycota	Sordariomycetes	Sordariales	Chaetomiaceae	NA	NA	1,00
**OTU146**	Fungi	Ascomycota	Sordariomycetes	Sordariales	Chaetomiaceae	*Chaetomium*	*Chaetomium longiciliata*	0,82
**OTU147**	Fungi	Ascomycota	Sordariomycetes	Sordariales	Chaetomiaceae	NA	NA	1,00
**OTU148**	Fungi	Ascomycota	Sordariomycetes	Hypocreales	Nectriaceae	*Fusarium*	*Fusarium neocosmosporiellum*	0,91
**OTU149**	Fungi	Ascomycota	Sordariomycetes	Sordariomycetes_ord_Incertae sedis	Thyridiaceae	*Pseudoneoconiothyrium*	*Pseudoneoconiothyrium rosae*	0,96
**OTU150**	Fungi	Ascomycota	Sordariomycetes	Glomerellales	Plectosphaerellaceae	*Chordomyces*	*Chordomyces antarcticus*	1,00
**OTU151**	Fungi	Ascomycota	Sordariomycetes	Hypocreales	Hypocreales_fam_Incertae_sedis	*Acremonium*	*Acremonium furcatum*	0,97
**OTU152**	Fungi	Ascomycota	Sordariomycetes	Glomerellales	Plectosphaerellaceae	*Plectosphaerella*	*Plectosphaerella cucumerina*	0,96
**OTU153**	Fungi	Ascomycota	Dothideomycetes	Pleosporales	Sporormiaceae	*Preussia*	*Preussia persica*	0,83
**OTU154**	Fungi	Ascomycota	Dothideomycetes	Pleosporales	Pleosporales_fam_Incertae_sedis	*Nigrograna*	NA	0,99
**OTU155**	Fungi	Ascomycota	Dothideomycetes	Pleosporales	Pleosporales_fam_Incertae_sedis	*Nigrograna*	*Nigrograna cangshanensis*	0,80
**OTU156**	Fungi	Ascomycota	Dothideomycetes	Pleosporales	Dictyosporiaceae	*Pseudoconiothyrium*	*Pseudoconiothyrium broussonetiae*	0,76

OTU	Kingdom	Phylum	Class	Order	Family	Genus	Species	Conf. *
**OTU157**	Fungi	Ascomycota	Dothideomycetes	Pleosporales	Phaeosphaeriaceae	*Phaeosphaeria*	NA	0,72
**OTU158**	Fungi	Ascomycota	Sordariomycetes	Diaporthales	Valsaceae	NA	NA	1,00
**OTU159**	Fungi	Ascomycota	Leotiomycetes	Thelebolales	Pseudeurotiaceae	*Pseudeurotium*	NA	0,94
**OTU160**	Fungi	Ascomycota	Leotiomycetes	Thelebolales	Pseudeurotiaceae	*Pseudeurotium*	NA	0,98
**OTU161**	Fungi	Ascomycota	Leotiomycetes	Thelebolales	Pseudeurotiaceae	*Pseudeurotium*	NA	0,71
**OTU162**	Fungi	Ascomycota	Eurotiomycetes	Eurotiales	Aspergillaceae	*Aspergillus*	*Aspergillus inflatus*	0,76
**OTU163**	Fungi	Ascomycota	Eurotiomycetes	Eurotiales	Aspergillaceae	*Aspergillus*	*Aspergillus inflatus*	1,00
**OTU164**	Fungi	Basidiomycota	Tremellomycetes	Filobasidiales	Piskurozymaceae	*Solicoccozyma*	NA	1,00
**OTU165**	Fungi	Ascomycota	Dothideomycetes	Pleosporales	Aigialaceae	*Posidoniomyces*	*Posidoniomyces atricolor*	1,00
**OTU166**	Fungi	Ascomycota	Eurotiomycetes	Onygenales	Onygenales_fam_Incertae_sedis	*Chrysosporium*	*Chrysosporium carmichaelii*	1,00
**OTU167**	Fungi	Ascomycota	Sordariomycetes	Sordariales	Lasiosphaeriaceae	*Podospora*	NA	0,90
**OTU168**	Fungi	Ascomycota	Sordariomycetes	Hypocreales	NA	NA	NA	0,93
**OTU169**	Fungi	Ascomycota	Dothideomycetes	Pleosporales	Dictyosporiaceae	*Pseudoconiothyrium*	*Pseudoconiothyrium broussonetiae*	1,00
**OTU170**	Fungi	Ascomycota	Dothideomycetes	Pleosporales	Pleosporaceae	*Alternaria*	NA	1,00
**OTU171**	Fungi	Ascomycota	Sordariomycetes	Hypocreomycetidae	Microascales	*Wardomycopsis*	NA	0,85
**OTU172**	Fungi	Ascomycota	Sordariomycetes	Microascales	Microascaceae	*Scedosporium*	Undefined Saprotroph	0,93
**OTU173**	Fungi	Ascomycota	Sordariomycetes	Hypocreales	Sarocladiaceae	*Parasarocladium*	*Parasarocladium alavariense*	0,92
**OTU174**	Fungi	Ascomycota	Pezizomycotina_cls_Incertae sedis	Pezizomycotina_ord_Incertae sedis	Pezizomycotina_fam_Incertae sedis	NA	NA	0,98
**OTU175**	Fungi	Ascomycota	Pezizomycotina_cls_Incertae sedis	Pezizomycotina_ord_Incertae sedis	Pezizomycotina_fam_Incertae sedis	NA	NA	1,00
**OTU176**	Fungi	Ascomycota	Sordariomycetes	Sordariales	NA	NA	NA	0,95
**OTU177**	Fungi	Ascomycota	Sordariomycetes	Glomerellales	Plectosphaerellaceae	*Gibellulopsis*	NA	1,00
**OTU178**	Fungi	Ascomycota	Dothideomycetes	Pleosporales	Dictyosporiaceae	*Pseudoconiothyrium*	NA	0,99
**OTU179**	Fungi	Ascomycota	Sordariomycetes	Hypocreales	Cordycipitaceae	*Beauveria*	NA	1,00
**OTU180**	Fungi	Ascomycota	Dothideomycetes	Pleosporales	NA	NA	NA	0,99
**OTU181**	Fungi	Ascomycota	Saccharomycetes	Saccharomycetales	Metschnikowiaceae	*Metschnikowia*	*Metschnikowia bicuspidata*	0,93
**OTU182**	Fungi	Ascomycota	Eurotiomycetes	Onygenales	Ajellomycetaceae	*Emmonsiellopsis*	*Emmonsiellopsis coralliformis*	0,72
**OTU183**	Fungi	Ascomycota	Eurotiomycetes	Onygenales	Gymnoascaceae	*Leucothecium*	NA	1,00

**OTU**	**Kingdom**	**Phylum**	**Class**	**Order**	**Family**	**Genus**	**Species**	**Conf. ***
**OTU184**	Fungi	Ascomycota	Eurotiomycetes	Eurotiales	Trichocomaceae	*Talaromyces*	*Talaromyces minioluteus*	0,99

Table S2. The 131 OTUs classified based on their putative function through FUNGuild.

Accession code	OTUs	Taxa	Functional Groups	Citation Source	Confidence Ranking	General Note
SH1155568,08FU_GU112754_reps_singleton	**OTU001**	*Clonostachys_rosea*	Plant Pathogen	Tedersoo et al. 2014	Probable	NA
SH1166461,08FU_JQ247388_reps_singleton	**OTU006**	*Penicillium*	Dung Saprotroph	Duncan & Eslyn 1966; Seehann et al. 1975; Costa et al. 2012; Bills et al. 2013; Tedersoo et al. 2014	Highly Probable	NA
SH1167465,08FU_KX507077_reps_singleton	**OTU007**	*Penicillium georgiense*	Dung Saprotroph	Duncan & Eslyn 1966; Seehann et al. 1975; Costa et al. 2012; Bills et al. 2013; Tedersoo et al. 2015	Highly Probable	NA
SH1167730,08FU_KT266856_reps_singleton	**OTU008**	*Penicillium adametzioides*	Dung Saprotroph	Duncan & Eslyn 1966; Seehann et al. 1975; Costa et al. 2012; Bills et al. 2013; Tedersoo et al. 2016	Highly Probable	NA
SH1168475,08FU_KJ413382_reps_singleton	**OTU010**	*Auxarthron chlamydosporum*	Undefined Saprotroph	Tedersoo et al. 2014	Probable	NA
SH1176727,08FU_KY768864_reps_singleton	**OTU011**	*Sparticola*	Dung Saprotroph	Cannon & Kirk, 2007	Probable	Saprobic, associated with dung and rotting vegetation (Cannon & Kirk 2007)
SH1180251,08FU_KM519637_reps_singleton	**OTU012**	*Candida norvegica*	Animal Pathogen	Manolakaki et al. 2010; Mekbib et al. 2011	Probable	This asexual genus is polyphyletic and contain species that function very differently depend on what they are. Caution should be taken when interpreting guilds for *Candida* species.
SH1183339,08FU_KU314944_reps_singleton	**OTU013**	*Roussoella*	Undefined Saprotroph	Tedersoo et al. 2014	Probable	NA
SH1186068,08FU_FJ528699_reps_singleton	**OTU014**	*Myriodontium keratinophilum*	Dung Saprotroph	Cannon & Kirk, 2007	Probable	Generally saprobic and keratinophilic, in soil or coprophilous, occasionally found on hair or horn (Cannon & Kirk 2007)
SH1231342,08FU_KP991454_reps_singleton	**OTU015**	*Preussia*	Undefined Saprotroph	Tedersoo et al. 2014	Probable	NA
SH1235309,08FU_GU053868_reps_singleton	**OTU016**	Phaeosphaeriaceae	Fungal Parasite	Cannon & Kirk, 2007	Probable	Necrotrophic or saprobic on a wide range of plants, especially monocotyledons; a few species parasitic on other fungi (Cannon & Kirk 2007)
SH1244566,08FU_HG937101_reps_singleton	**OTU018**	*Gibellulopsis*	Plant Pathogen	Tedersoo et al. 2014	Probable	NA
SH1664879,08FU_UDB0200699_reps	**OTU020**	*Pseudoconiothyrium broussonetiae*	Plant Pathogen	Põlme et al. 2020	Probable	NA
SH1667325,08FU_UDB083980_reps	**OTU022**	*Coprinellus verrucispermus*	Undefined Saprotroph	Tedersoo et al. 2014	Probable	NA
SH1669319,08FU_MT603917_reps	**OTU023**	*Rhizopus arrhizus*	Plant Pathogen	Tedersoo et al. 2014	Probable	NA
SH1669648,08FU_LM652407_refs	**OTU024**	*Microascus*	Animal Pathogen	James et al. 2006; Costa et al. 2012; Bills et al. 2013; Irinyi et al. 2015	Possible	NA

Accession code	OTUs	Taxa	Functional Groups	Citation Source	Confidence Ranking	General Note
SH1670937,08FU_JX235700_refs	**OTU025**	*Westerdykella reniformis*	Undefined Saprotroph	Tedersoo et al. 2014	Probable	NA
SH1672642,08FU_MH855821_reps	**OTU026**	*Graphium*	Animal Pathogen	Duncan & Eslyn 1966; Irinyi et al. 2015; Lawrey & Diederich 2016; Tedersoo et al. 2014	Probable	Some (e.g., *G. basitruncatum*) likely opportunistic human pathogen (Irinyi et al. 2015)
SH1673021,08FU_MK018804_reps	**OTU027**	*Acremonium spinosum*	Animal Pathogen	Duncan & Eslyn WE. 1966; Nilsson 1973; Worrall et al. 1997; Hanada et al. 2010; Del Olmo-Ruiz & Arnold, 2014; Tedersoo et al. 2014; Irinyi et al. 2015	Possible	Endophyte detection method-culture (Hanada et al. 2010, Del Olmo-Ruiz & Arnold 2014); Likely opportunistic human pathogen (Irinyi et al. 2015)
SH1676182,08FU_HQ605705_refs	**OTU028**	*Talaromyces albobiverticillius*	Undefined Saprotroph	Tedersoo et al. 2014	Probable	NA
SH1676193,08FU_JN899394_refs	**OTU029**	*Talaromyces ucrainicus*	Undefined Saprotroph	Tedersoo et al. 2014	Probable	NA
SH1676208,08FU_MK018741_reps	**OTU030**	*Talaromyces clemensii*	Undefined Saprotroph	Tedersoo et al. 2014	Probable	NA
SH1676231,08FU_MH857885_refs	**OTU031**	*Talaromyces rubicundus*	Undefined Saprotroph	Tedersoo et al. 2014	Probable	NA
SH1676465,08FU_UDB0768742_reps	**OTU032**	*Talaromyces rubicundus*	Undefined Saprotroph	Tedersoo et al. 2014	Probable	NA
SH1677080,08FU_KY852475_refs	**OTU034**	*Kernia columnaris*	Dung Saprotroph	Bills et al. 2013; Tedersoo et al. 2014	Highly Probable	NA
SH1678407,08FU_AF486131_refs	**OTU035**	*Phialocephala humicola*	Endophyte	Newsham, 2011	Highly Probable	Dark Septate Endophyte, known from plant roots.
SH1678835,08FU_AJ292420_refs	**OTU036**	*Acrostalagmus luteoalbus*	Endophyte	Costa et al. 2012; Tedersoo et al. 2014	Probable	NA
SH1684017,08FU_DQ123588_reps	**OTU037**	*Clonostachys*	Algal Parasite	Cannon & Kirk PM. 2007	Probable	NA
SH1685984,08FU_HQ880789_refs	**OTU038**	*Beauveria*	Animal Pathogen	Tedersoo et al. 2014	Probable	NA
SH1686716,08FU_UDB015413_refs	**OTU039**	*Phallus impudicus*	Litter Saprotroph	Dix & Cairney 1985; Grgurinovic & Simpson 2001; Cannon & Kirk PM. 2007; Tedersoo L, et al. 2014	Probable	Common name - stinkhorn; Saprobic, on soil or decaying wood, with spores dispersed by insects that are attracted to the fruit bodies by their distinctive foetid smell (Cannon & Kirk 2007)
SH1686824,08FU_JF740262_refs	**OTU040**	*Pyrenochaetopsis leptospora*	Endophyte	Tedersoo et al. 2014	Possible	NA
SH1687333,08FU_MG828932_reps	**OTU041**	*Paraconiothyrium*	Endophyte	Cannon & Kirk PM. 2007; Massimo et al. 2015	Probable	Saprobes in both woody and herbaceous material, also parasitic on other fungi (Cannon & Kirk 2007)
SH1687356,08FU_GU946458_reps	**OTU042**	*Paraconiothyrium variabile*	Undefined Saprotroph	Tedersoo et al. 2014	Probable	NA

Accession code	OTUs	Taxa	Functional Groups	Citation Source	Confidence Ranking	General Note
SH1688438,08FU_KF156010_refs	**OTU043**	*Paradendryphiella arenariae*	Plant Pathogen	Tedersoo et al. 2014	Probable	NA
SH1689080,08FU_MH861939_refs	**OTU044**	*Alternaria*	Animal Pathogen	Duncan & Eslyn 1966; Seehann et al. 1975; Tedersoo et al. 2014	Possible	NA
SH1689759,08FU_KC460874_reps	**OTU045**	*Coniophora*	Wood Saprotroph	Tedersoo et al. 2014	Probable	NA
SH1689902,08FU_MW218880_reps	**OTU046**	*Corollospora*	Undefined Saprotroph	Tedersoo et al. 2014	Probable	NA
SH1692774,08FU_AF033475_refs	**OTU048**	*Penicillium nalgiovense*	Dung Saprotroph	Duncan & Eslyn 1966; Seehann et al. 1975; Costa et al. 2012; Bills et al. 2013; Tedersoo et al. 2014	Highly Probable	NA
SH1692782,08FU_DQ645805_refs	**OTU051**	*Penicillium brevicompactum*	Dung Saprotroph	Duncan & Eslyn 1966; Seehann et al. 1975; Costa et al. 2012; Bills et al. 2013; Tedersoo et al. 2014	Highly Probable	NA
SH1692794,08FU_AF037431_refs	**OTU052**	*Penicillium pimiteouiense*	Dung Saprotroph	Duncan & Eslyn 1966; Seehann et al. 1975; Costa et al. 2012; Bills et al. 2013; Tedersoo et al. 2014	Highly Probable	NA
SH1699137,08FU_UDB0766196_reps	**OTU055**	*Penicillium*	Dung Saprotroph	Duncan & Eslyn 1966; Seehann et al. 1975; Costa et al. 2012; Bills et al. 2013; Tedersoo et al. 2014	Highly Probable	NA
SH1699529,08FU_MH935985_reps	**OTU057**	*Penicillium menonorum*	Dung Saprotroph	Duncan & Eslyn 1966; Seehann et al. 1975; Costa et al. 2012; Bills et al. 2013; Tedersoo et al. 2014	Highly Probable	NA
SH1699989,08FU_JN376148_reps	**OTU058**	*Penicillium ubiquetum*	Dung Saprotroph	Duncan & Eslyn 1966; Seehann et al. 1975; Costa et al. 2012; Bills et al. 2013; Tedersoo et al. 2014	Highly Probable	NA
SH1700040,08FU_MG333472_reps	**OTU059**	*Penicillium*	Dung Saprotroph	Duncan & Eslyn 1966; Seehann et al. 1975; Costa et al. 2012; Bills et al. 2013; Tedersoo et al. 2014	Highly Probable	NA
SH1700462,08FU_UDB0768749_reps	**OTU060**	*Penicillium*	Dung Saprotroph	Duncan & Eslyn 1966; Seehann et al. 1975; Costa et al. 2012; Bills et al. 2013; Tedersoo et al. 2014	Highly Probable	NA
SH1701968,08FU_MH859492_refs	**OTU062**	*Gymnoascus udagawae*	Undefined Saprotroph	Tedersoo et al. 2014	Probable	NA
SH1703422,08FU_MG938463_reps	**OTU063**	*Arthrinium*	Undefined Saprotroph	Tedersoo et al. 2014	Probable	NA
SH1703441,08FU_KT385730_reps	**OTU064**	*Arthrinium rasikravindrae*	Undefined Saprotroph	Tedersoo et al. 2014	Probable	NA
SH1705669,08FU_KP101211_reps	**OTU065**	*Sporormiella minimoides*	Undefined Saprotroph	Tedersoo et al. 2014	Probable	NA
SH1708019,08FU_HE974452_refs	**OTU066**	*Auxarthron ostraviense*	Undefined Saprotroph	Tedersoo et al. 2014	Probable	NA

Accession code	OTUs	Taxa	Functional Groups	Citation Source	Confidence Ranking	General Note
SH1710543,08FU_HE610364_refs	**OTU067**	*Phialemonium inflatum*	Undefined Saprotroph	Tedersoo et al. 2014	Probable	NA
SH1711294,08FU_KF986412_reps	**OTU068**	*Ascotricha erinacea*	Undefined Saprotroph	Tedersoo et al. 2014	Probable	NA
SH1711388,08FU_MG250173_refs	**OTU069**	*Paracremonium*	Undefined Saprotroph	Tedersoo et al. 2014	Probable	NA
SH1711434,08FU_FJ752600_reps	**OTU070**	*Fusarium nematophilum*	Plant Pathogen	Cannon & Kirk 2007; Hirooka Y, et al. 2012; Tedersoo et al. 2014	Possible	*Fusarium* species are amongst the economically significant plant pathogens (Cannon & Kirk 2007)
SH1711640,08FU_MH855187_reps	**OTU071**	*Rectifusarium ventricosum*	Plant Pathogen	Duncan & Eslyn 1966; Seehann et al. 1975; Costa et al. 2012; Tedersoo et al. 2014; Irinyi et al. 2015; Schroers et al. 2016; Lawrey & Diederich, 2016	Possible	Likely opportunistic human pathogen (Irinyi et al. 2015)
SH1712486,08FU_MN904866_reps	**OTU072**	*Didymella*	Animal Pathogen	Cannon & Kirk 2007; Hirooka Y, et al. 2012; Tedersoo et al. 2014	Possible	*Fusarium* species are amongst the economically significant plant pathogens (Cannon & Kirk 2007)
SH1712518,08FU_MT816429_reps	**OTU073**	Didymellaceae	Animal Pathogen	Tedersoo et al. 2014	Possible	NA
SH1715004,08FU_GQ303268_refs	**OTU074**	*Antennariella placitae*	Animal Pathogen	Tedersoo et al. 2015	Probable	NA
SH1715275,08FU_AB734456_reps	**OTU075**	Gymnoascaceae	Undefined Saprotroph	Cannon & Kirk 2007	Probable	Keratinophilic or cellulolytic, usually encountered as isolations from soil, dung, etc. (Cannon & Kirk 2007)
SH1717816,08FU_KT715718_reps	**OTU078**	*Brunneofusispora sinensis*	Wood Saprotroph	Põlme et al. 2020	Probable	NA
SH1717823,08FU_MT159630_reps	**OTU080**	*Brunneofusispora sinensis*	Wood Saprotroph	Põlme et al. 2020	Probable	NA
SH1719385,08FU_MK613177_reps	**OTU083**	*Trichoderma*	Plant Pathogen	Seehann et al. 1975; Andrews et al. 1983; Hanada et al. 2010; Costa et al. 2012; Tedersoo et al. 2014; Irinyi et al. 2015; Busby et al. 2016	Probable	Cause of soft-rot (Seehann et al. 1975); likely opportunistic human pathogen (Irinyi et al. 2015); endophyte detection method-culture (Busby et al. 2016)
SH1721447,08FU_KT699144_reps	**OTU086**	*Plectosphaerellaceae*	Animal Pathogen	Duncan & Eslyn 1966; Nilsson 1973; Worrall et al. 1997; Hanada et al. 2010; Del Olmo-Ruiz& Arnold 2014; Tedersoo et al. 2014; Irinyi et al. 2015	Possible	NA
SH1722884,08FU_AM901798_reps	**OTU087**	*Monographella nivalis*	Plant Pathogen	Tedersoo et al. 2014	Probable	NA
SH1722973,08FU_KP257156_refs	**OTU088**	*Hodophilus variabilipes*	Undefined Saprotroph	Cannon & Kirk 2007; Tedersoo et al. 2014	Probable	Most species are presumed saprobic on the ground or rotten wood, but many *Multiclavula* species are lichenized with green algae (Cannon & Kirk 2007)

Accession code	OTUs	Taxa	Functional Groups	Citation Source	Confidence Ranking	General Note
SH1724102,08FU_DQ249194_refs	**OTU089**	*Candida norvegica*	Animal Pathogen	Manolakaki et al. 2010; Mekbib et al. 2011	Probable	This asexual genus is polyphyletic and contain species that function very differently. Caution should be taken when interpreting guilds for *Candida* species.
SH1724132,08FU_DQ112599_refs	**OTU090**	*Bovista*	Bryophyte Parasite	Kirk et al. 2008	Possible	Mushrooms and toadstools, Gill fungi, Agarics; lignicolous, saprobic, mycorrhizal, rarely parasitic on plants or fungi (Kirk et al. 2008)
SH1724148,08FU_DQ112621_refs	**OTU091**	*Bovista aestivalis*	Soil Saprotroph	Grgurinovic & Simpson 2001; Tedersoo et al. 2014	Probable	NA
SH1724917,08FU_MK018239_reps	**OTU093**	*Geomyces*	Wood Saprotroph	Cannon & Kirk PM. 2007	Probable	Saprobic on woody tissue and rotting vegetation, often isolated from soil (Cannon & Kirk 2007)
SH1725573,08FU_DQ006923_reps	**OTU094**	*Eutypa tetragona*	Plant Pathogen	Tedersoo et al. 2014	Probable	NA
SH1729406,08FU_KU059879_reps	**OTU096**	*Hypomyces*	Fungal Parasite	List compiled by John Plischke III	Highly Probable	Sexual form with asexual forms spread across multiple genera
SH1729460,08FU_KU255069_reps	**OTU097**	*Lophiostoma*	Undefined Saprotroph	Tedersoo et al. 2014	Probable	NA
SH1729967,08FU_KU846295_refs	**OTU098**	*Myrothecium roridum*	Undefined Saprotroph	Tedersoo et al. 2014	Probable	NA
SH1730423,08FU_KY434121_reps	**OTU100**	*Roussoella*	Undefined Saprotroph	Tedersoo et al. 2014	Probable	NA
SH1730426,08FU_KU255054_reps	**OTU101**	*Roussoella neopustulans*	Undefined Saprotroph	Tedersoo et al. 2014	Probable	NA
SH1730428,08FU_KU314951_reps	**OTU102**	*Parathyridaria*	Wood Saprotroph	Cannon & Kirk PM. 2007	Probable	NA
SH1730429,08FU_KR014367_reps	**OTU103**	*Parathyridaria*	Wood Saprotroph	Cannon & Kirk PM. 2008	Probable	NA
SH1730470,08FU_EU142849_reps	**OTU104**	*Penicillium*	Dung Saprotroph	Duncan & Eslyn 1966; Seehann et al. 1975; Costa et al. 2012; Bills et al. 2013; Tedersoo et al. 2014	Highly Probable	NA
SH1730475,08FU_MT821161_reps	**OTU105**	*Penicillium*	Dung Saprotroph	Duncan & Eslyn 1966; Seehann et al. 1975; Costa et al. 2012; Bills et al. 2013; Tedersoo et al. 2014	Highly Probable	NA
SH1730613,08FU_UDB0764227_reps	**OTU106**	*Volutella*	Animal Pathogen	Cannon & Kirk PM. 2007; Hirooka et al. 2012; Tedersoo et al. 2014	Possible	NA
SH1733825,08FU_KC412721_reps	**OTU107**	*Lulwoana*	Undefined Saprotroph	Tedersoo et al. 2014	Probable	NA
SH1733826,08FU_KF915992_reps	**OTU108**	*Lulwoana*	Undefined Saprotroph	Tedersoo et al. 2014	Probable	NA
SH1734181,08FU_MK018717_reps	**OTU109**	*Xylogone*	Fungal Parasite	Tedersoo et al. 2014	Probable	NA

Accession code	OTUs	Taxa	Functional Groups	Citation Source	Confidence Ranking	General Note
SH1734301,08FU_MK018793_reps	**OTU110**	*Scytalidium*	Wood Saprotroph	Nilsson 1973; Costa et al. 2012, Tedersoo et al. 2014	Probable	NA
SH1734711,08FU_AY498676_reps	**OTU111**	*Phaeophyscia orbicularis*	Lichenized	Esslinger, 2014	Highly Probable	NA
SH1735926,08FU_FJ865442_reps	**OTU112**	*Fomes inzengae*	Wood Saprotroph	Gilbertson & Ryvarden 1987-1987	Highly Probable	NA
SH1737792,08FU_MK018251_reps	**OTU113**	*Albifimbria*	Fungal Parasite	Lombard & et al. 2016	Probable	NA
SH1738437,08FU_AF443920_refs	**OTU114**	*Trichoderma*	Animal Pathogen	Andrews et al. 1983; Hanada et al. 2010; Costa et al. 2012; Tedersoo et al. 2014; Irinyi et al. 2015; Busby et al. 2016	Probable	Likely opportunistic human pathogen (Irinyi et al. 2015); endophyte detection method-culture (Busby et al. 2016)
SH1738438,08FU_DQ677655_refs	**OTU115**	*Trichoderma koningii*	Animal Pathogen	Andrews et al. 1983; Hanada et al. 2010; Costa et al. 2012; Tedersoo et al. 2014; Irinyi et al. 2015; Busby et al. 2017	Probable	Likely opportunistic human pathogen (Irinyi et al. 2015); endophyte detection method-culture (Busby et al. 2016)
SH1739088,08FU_FJ860721_refs	**OTU116**	*Trichoderma albolutescens*	Animal Pathogen	Andrews et al. 1983; Hanada et al. 2010; Costa et al. 2012; Tedersoo et al. 2014; Irinyi et al. 2015; Busby et al. 2018	Probable	Likely opportunistic human pathogen (Irinyi et al. 2015); endophyte detection method-culture (Busby et al. 2016)
SH1739207,08FU_MH865819_refs	**OTU117**	*Trichoderma*	Animal Pathogen	Andrews et al. 1983; Hanada et al. 2010; Costa et al. 2012; Tedersoo et al. 2014; Irinyi et al. 2015; Busby et al. 2019	Probable	Likely opportunistic human pathogen (Irinyi et al. 2015); endophyte detection method-culture (Busby et al. 2016)
SH1739555,08FU_GU131272_reps	**OTU118**	*Trichoderma koningii*	Animal Pathogen	Andrews et al. 1983; Hanada et al. 2010; Costa et al. 2012; Tedersoo et al. 2014; Irinyi et al. 2015; Busby et al. 2020	Probable	Likely opportunistic human pathogen (Irinyi et al. 2015); endophyte detection method-culture (Busby et al. 2016)
SH1740220,08FU_JX570932_refs	**OTU119**	*Biatriospora antibiotica*	Animal Pathogen	Kolařík et al. 2017	Probable	Saprotroph isolated from decaying wood in marine habitats-an opportunistic human pathogen (Kolařík et al. 2017)
SH1740469,08FU_KY026600_refs	**OTU120**	*Aphanoascus keratinophilus*	Undefined Saprotroph	Tedersoo et al. 2014	Probable	NA
SH1740470,08FU_AB361656_reps	**OTU121**	*Aphanoascus keratinophilus*	Undefined Saprotroph	Tedersoo et al. 2014	Probable	NA
SH1741055,08FU_UDB0258307_reps	**OTU123**	*Acremonium hennebertii*	Animal Pathogen	Duncan & Eslyn 1966; Nilsson 1973; Hanada et al. 2010; Del Olmo-Ruiz & Arnold, 2014; Tedersoo et al. 2014; Irinyi et al. 2015	Possible	NA
SH1743105,08FU_UDB0311602_reps	**OTU124**	*Pseudaleuria*	Undefined Saprotroph	Rinaldi et al. 2008; Tedersoo et al. 2010	Possible	NA
SH1743904,08FU_MW090878_reps	**OTU125**	*Eutypa flavovirens*	Plant Pathogen	Tedersoo et al. 2014	Probable	NA

Accession code	OTUs	Taxa	Functional Groups	Citation Source	Confidence Ranking	General Note
SH1745143,08FU_AB809645_refs	**OTU131**	*Periconia*	Endophyte	Duncan & Eslyn 1966; Costa et al. 2012; Mandyam et al. 2012; Knapp et al. 2012	Possible	Dark Septate Endophyte, known from plant roots (Mandyam et al. 2010); endophyte host - Asteraceae, Musaceae (Costa et al. 2012)
SH1746151,08FU_KU325128_reps	**OTU132**	*Heydenia alpina*	Dung Saprotroph	Cannon & Kirk PM. 2007	Possible	Saprobic on soil, dung or rotten wood or mycorrhizal, some species hypogeous, others associated with fire sites (Cannon & Kirk 2007)
SH1748112,08FU_LN850960_refs	**OTU133**	*Cephalotrichum asperulum*	Undefined Saprotroph	Cannon & Kirk PM. 2007	Possible	Saprobic in soil or rotting vegetation, some species heat-tolerant and associated with silage and compost, a few associated with humans and animals (Cannon & Kirk 2007)
SH1748115,08FU_LM652379_refs	**OTU134**	*Gamsia simplex*	Undefined Saprotroph	Cannon & Kirk PM. 2007	Possible	Saprobic in soil or rotting vegetation, some species heat-tolerant and associated with silage and compost, a few associated with humans and animals (Cannon & Kirk 2007)
SH1773167,08FU_MK142270_reps	**OTU135**	*Metarhizium robertsii*	Animal Pathogen	Tedersoo et al. 2014	Probable	NA
SH1773219,08FU_AB540572_refs	**OTU136**	*Acremonium dichromosporum*	Animal Pathogen	Duncan & Eslyn 1966; Costa et al. 2012; Mandyam et al. 2012; Knapp et al. 2012	Possible	Endophyte detection method-culture (Hanada et al. 2010; Del Olmo-Ruiz & Arnold 2014); Likely opportunistic human pathogen (Irinyi et al. 2015)
SH1787736,08FU_AY328915_refs	**OTU137**	*Wallemia sebi*	Undefined Saprotroph	Tedersoo et al. 2014	Probable	NA
SH1787753,08FU_MK018974_reps	**OTU138**	*Wallemia*	Undefined Saprotroph	Tedersoo et al. 2014	Probable	NA
SH1788553,08FU_JQ905732_reps	**OTU139**	*Acremonium cavaraeanum*	Animal Pathogen	Duncan & Eslyn 1966; Costa et al. 2012; Mandyam et al. 2012; Knapp et al. 2012	Possible	Endophyte detection method-culture (Hanada et al. 2010; Del Olmo-Ruiz & Arnold 2014); Likely opportunistic human pathogen (Irinyi et al. 2015)
SH1799110,08FU_KF251149_refs	**OTU141**	*Setophaeosphaeria badalingensis*	Fungal Parasite	Cannon & Kirk PM. 2007	Probable	Necrotrophic or saprobic on a wide range of plants, especially monocotyledons; a few species parasitic on other fungi (Cannon & Kirk 2007)
SH1799113,08FU_MK019111_reps	**OTU142**	*Pyrenochaeta*	Wood Saprotroph	Cannon & Kirk PM. 2007	Probable	Necrotrophic or saprobic on woody plants (Cannon & Kirk 2007)
SH1800595,08FU_KX976601_refs	**OTU143**	*Botryotrichum spirotrichum*	Undefined Saprotroph	Tedersoo et al. 2014	Probable	NA
SH1810918,08FU_KY379732_reps	**OTU148**	*Fusarium neocosmosporiellum*	Plant Pathogen	Duncan & Eslyn 1966; Costa et al. 2012; Mandyam et al. 2012; Knapp et al. 2012	Possible	Likely opportunistic human pathogen (Irinyi et al. 2015)
SH1836219,08FU_KJ443241_refs	**OTU150**	*Chordomyces antarcticus*	Animal Pathogen	Duncan & Eslyn 1966; Costa et al. 2012; Mandyam et al. 2012; Knapp et al. 2012	Possible	NA
SH1836220,08FU_AY378154_refs	**OTU151**	*Acremonium furcatum*	Animal Pathogen	Duncan & Eslyn 1966; Costa et al. 2012; Mandyam et al. 2012; Knapp et al. 2012	Possible	Endophyte detection method-culture (Hanada et al. 2010; Del Olmo-Ruiz & Arnold 2014); Likely opportunistic human pathogen (Irinyi et al. 2015)

Accession code	OTUs	Taxa	Functional Groups	Citation Source	Confidence Ranking	General Note
SH1836405,08FU_UDB0764621_reps	**OTU152**	*Plectosphaerella cucumerina*	Endophyte	Palm et al. 1995; Carlucci et al. 2012; David et al. 2016; Li et al. 2017	Probable	*Plectosporium* was introduced by Palm et al. (1995) for *Fusarium tabacinum*, the anamorph of *Plectosphaerella cucumerina* (Carlucci et al. 2012)
SH1836220,08FU_AY378154_refs	**OTU151**	*Acremonium furcatum*	Animal Pathogen	Duncan & Eslyn 1966; Costa et al. 2012; Mandyam et al. 2012; Knapp et al. 2012	Possible	Endophyte detection method-culture (Hanada et al. 2010; Del Olmo-Ruiz & Arnold 2014); Likely opportunistic human pathogen (Irinyi et al. 2015)
SH1836405,08FU_UDB0764621_reps	**OTU152**	*Plectosphaerella cucumerina*	Endophyte	Palm et al. 1995; Carlucci et al. 2012; David et al. 2016; Li et al. 2017	Probable	*Plectosporium* was introduced by Palm et al. (1995) for *Fusarium tabacinum*, the anamorph of *Plectosphaerella cucumerina* (Carlucci et al. 2012)
SH1838138,08FU_KP335547_reps	**OTU153**	*Preussia persica*	Undefined Saprotroph	Tedersoo et al. 2014	Probable	NA
SH1838153,08FU_KC339242_reps	**OTU154**	*Nigrograna*	Animal Pathogen	Tedersoo et al. 2014	Probable	NA
SH1838283,08FU_HQ207026_reps	**OTU155**	*Nigrograna cangshanensis*	Animal Pathogen	Tedersoo et al. 2014	Probable	NA
SH1838568,08FU_UDB0184192_reps	**OTU156**	*Pseudoconiothyrium broussonetiae*	Plant Pathogen	Põlme et al. 2020	Probable	NA
SH1838685,08FU_KU933733_reps	**OTU157**	*Phaeosphaeria*	Undefined Saprotroph	Tedersoo et al. 2014	Probable	NA
SH1839405,08FU_KC342485_reps	**OTU158**	Valsaceae	Undefined Saprotroph	Cannon & Kirk PM. 2007	Probable	Saprobes, endophytes and weak to virulent parasites of woody plants (Cannon & Kirk 2007)
SH1840209,08FU_AY129291_refs	**OTU159**	*Pseudeurotium*	Undefined Saprotroph	Tedersoo et al. 2014	Probable	NA
SH1840223,08FU_FN397281_reps	**OTU160**	*Pseudeurotium*	Undefined Saprotroph	Tedersoo et al. 2014	Probable	NA
SH1840226,08FU_AB520862_reps	**OTU161**	*Pseudeurotium*	Undefined Saprotroph	Tedersoo et al. 2014	Probable	NA
SH2592636,08FU_MK442618_reps_singleton	**OTU169**	*Pseudoconiothyrium broussonetiae*	Plant Pathogen	Põlme et al. 2020	Probable	NA
SH2595961,08FU_MF156029_reps_singleton	**OTU170**	*Alternaria*	Wood Saprotroph	Duncan & Eslyn 1966; Costa et al. 2012; Mandyam et al. 2012; Knapp et al. 2012	Possible	NA
SH2763430,08FU_MT876169_reps	**OTU172**	*Scedosporium*	Undefined Saprotroph	Tedersoo et al. 2014	Probable	NA
SH2785894,08FU_UDB0571658_reps	**OTU177**	*Gibellulopsis*	Plant Pathogen	Tedersoo et al. 2014	Probable	NA
SH2790353,08FU_MK312328_reps	**OTU179**	*Beauveria*	Animal Pathogen	Tedersoo et al. 2014	Probable	NA

**Accession code**	**OTUs**	**Taxa**	**Functional Groups**	**Citation Source**	**Confidence Ranking**	**General Note**
SH3565214,08FU_LR598892_reps_singleton	**OTU182**	*Emmonsiellopsis coralliformis*	Undefined Saprotroph	Cannon & Kirk 2007	NA	NA
SH3566175,08FU_MK018334_reps_singleton	**OTU183**	*Leucothecium*	Undefined Saprotroph	Tedersoo et al. 2014	Probable	Saprobic phases colonizing soil, dung, dead wood etc., especially in arid conditions (Cannon & Kirk 2007)
SH3566382,08FU_MT441589_reps_singleton	**OTU184**	*Talaromyces minioluteus*	Undefined Saprotroph	Tedersoo et al. 2014	Probable	NA
